# P08 Antimicrobial drug efficacy in surgical site infections post soft tissue augmentation: an analytic review

**DOI:** 10.1093/jacamr/dlae136.012

**Published:** 2024-08-23

**Authors:** Abiola Odeyinka

**Affiliations:** University Hospital Derby and Burton NHS Trust, UK

## Abstract

**Background:**

Soft tissue augmentation refers to the process of adding volume, shape or contour to areas of the body where there is a deficiency or loss of soft tissue. Soft tissue augmentation is a common procedure for facial and body rejuvenation and enhancement and is often done using hyaluronic acid dermal fillers and calcium hydroxyapatite fillers and silicone implants. These fillers are popular due to their efficacy, durability and minimal invasiveness. For instance, a pilot study that examined the anti-ageing efficacy, tolerability and effects on skin quality of hyaluronic acid injections for the mid and lower face added to the evidence supporting the efficacy of these treatments in reducing signs of ageing and improving overall skin appearance. Whilst soft tissue augmentation procedures are termed as surgical procedures that carry minor risk of complications, post-operative infections are however very common and can often be difficult to treat due to antimicrobial resistance.

**Objectives:**

To understand how antimicrobials treat surgical site infections in soft tissue augmentation.

**Methods:**

This study evaluated the treatment outcomes of soft tissue augmentation using hyaluronic acid dermal fillers in patients, including anti-ageing efficacy, tolerability, skin hydration and elasticity. A combination of clinical assessments, instrumental techniques, and subjective evaluations was used to assess the efficacy of hyaluronic acid dermal fillers in the mid and lower face. The results showed high patient satisfaction, longer-lasting effects compared with other filler materials, and low incidence of immunogenic reaction due to the biodegradability and natural presence of hyaluronic acid in the human body.

**Results:**

All responses to the survey questions are presented as counts and percentages of the total sample to allow for a thorough understanding of how healthcare providers typically manage delayed inflamed reactions following the injection of hyaluronic acid dermal fillers. The survey results indicated a wide variation in healthcare providers’ experiences and understanding of managing delayed inflammatory reactions from hyaluronic acid dermal fillers. The results are presented in a contingency table. Differences in the distribution of responses between the study groups were evaluated by the χ^2^ test followed by pairwise comparisons with Benjamini-Hochberg correction. The significance level was defined as α=0.05. Of the 1120 practitioners who were sent the questionnaire, 334 (29.8%) responded, and their answers were analysed. The majority of the respondents were dentists (*n*=104, 31%), followed by dermatologists and plastic surgeons (*n*=63, 19%, and *n*=40, 12%, respectively), and other physicians (*n*=127, 38%). The study revealed a significant lack of standardized treatment practices and varying levels of experience among healthcare providers in managing delayed inflammatory reactions to hyaluronic acid dermal fillers.

**Conclusions:**

β-Lactam antibiotics, vancomycin, linezolid and clindamycin are frequently used to-treat surgical skin infections brought on by Gram-positive cocci. β-Lactam medicines are the cornerstone therapy for MSSA and suspected streptococcal infection. While soft tissue facial and body augmentation is desired for aesthetics purposes, there is a potential for serious complications; including surgical site infections, immunoreactions, serious vascular complications and necrotizing soft tissue infections, underscoring the necessity for careful administration and post-treatment monitoring of signs and symptoms of skin infections. Analysing treatment outcomes in soft tissue facial and body augmentation surgical procedures is crucial to determine the effectiveness and safety of these procedures and identify any potential areas for improvement.

